# Alterations in the kallikrein-kinin system predict death after heart transplant

**DOI:** 10.1038/s41598-022-18573-2

**Published:** 2022-08-19

**Authors:** Nicholas P. Giangreco, Guillaume Lebreton, Susan Restaino, Maryjane Farr, Emmanuel Zorn, Paolo C. Colombo, Jignesh Patel, Rajesh Kumar Soni, Pascal Leprince, Jon Kobashigawa, Nicholas P. Tatonetti, Barry M. Fine

**Affiliations:** 1 Departments of Systems Biology, Biomedical Informatics, and Medicine, Columbia University, New York, NY USA; 2 Chirurgie Thoracique et Cardiovasculaire, Pitíe-Salpetriere University Hospital, Paris, France; 3 Department of Medicine, Division of Cardiology, Columbia University Irving Medical Center, New York, NY USA; 4 Department of Medicine, Division of Cardiology, University of Texas Southwestern Medical Center, Dallas, TX USA; 5 Center for Translational Immunology, Columbia University Irving Medical Center, New York, NY USA; 6 Cedars-Sinai Heart Institute, Cedars Sinai Medical Center, Los Angeles, CA USA; 7 Proteomics and Macromolecular Crystallography Shared Resource, Herbert Irving Comprehensive Cancer Center, Columbia University Irving Medical Center, New York, NY USA; 8 Institute for Genomic Medicine, Columbia University, New York, NY USA

**Keywords:** Predictive markers, Prognostic markers

## Abstract

Heart transplantation remains the definitive treatment for end stage heart failure. Because availability is limited, risk stratification of candidates is crucial for optimizing both organ allocations and transplant outcomes. Here we utilize proteomics prior to transplant to identify new biomarkers that predict post-transplant survival in a multi-institutional cohort. Microvesicles were isolated from serum samples and underwent proteomic analysis using mass spectrometry. Monte Carlo cross-validation (MCCV) was used to predict survival after transplant incorporating select recipient pre-transplant clinical characteristics and serum microvesicle proteomic data. We identified six protein markers with prediction performance above AUROC of 0.6, including Prothrombin (F2), anti-plasmin (SERPINF2), Factor IX, carboxypeptidase 2 (CPB2), HGF activator (HGFAC) and low molecular weight kininogen (LK). No clinical characteristics demonstrated an AUROC > 0.6. Putative biological functions and pathways were assessed using gene set enrichment analysis (GSEA). Differential expression analysis identified enriched pathways prior to transplant that were associated with post-transplant survival including activation of platelets and the coagulation pathway prior to transplant. Specifically, upregulation of coagulation cascade components of the kallikrein-kinin system (KKS) and downregulation of kininogen prior to transplant were associated with survival after transplant. Further prospective studies are warranted to determine if alterations in the KKS contributes to overall post-transplant survival.

## Introduction

Heart transplantation remains an important treatment for end stage heart failure. Though the prevalence of heart failure continues to grow, the availability of suitable grafts remains static resulting in an allocation system predicated on severity of illness and wait list mortality risk^1^. Predicting survival is one potential avenue for improving graft utilization and balancing the risks of the waitlist against post-transplant mortality. Outcome prediction has the benefits of both applying evidence-based data to organ allocation and to improve the risk–benefit analysis of heart transplant in high-risk patients. Such tools have shown the ability to accurately classify post-transplant risk in other solid organ such as kidney and lung transplant^2,3^. Though biomarker based risk scores for heart failure have demonstrated acceptable performance metrics for prediction of wait list mortality, post-transplant prediction has demonstrated poor discrimination in external validation sets^4,5^. The result has been a lack of uniform or widespread adoption of any risk prediction tool post-transplant.

**Figure 1:**
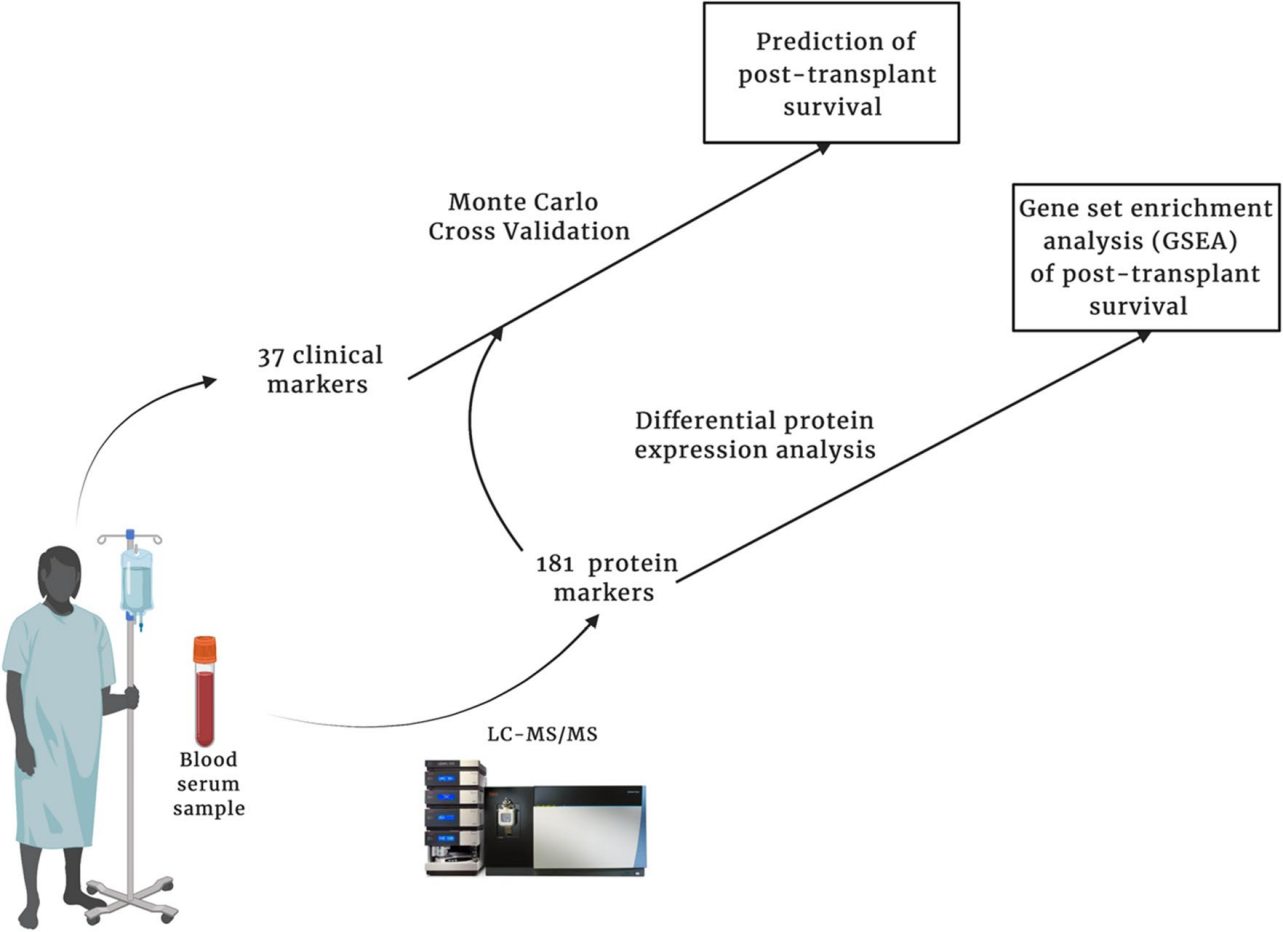
Study overview. Patient’s blood were drawn and serum was processed according to protocol at the clinical site (see “Methods”). We employed Monte Carlo Cross Validation (MCCV; See “Methods” for complete algorithm) to quantify a prediction interval of all pre-transplant, recipient markers to predict patient survival after heart transplantation. Additionally, the eligible protein markers were used in a differential protein expression analysis. Figure made with Biorender.

Circulating microvesicles are small membrane enclosed vesicles that contain protein, RNA and DNA and are part of a vast intercellular communication network found in all bodily fluids^6^. The microvesicle proteome serves as a rich resource of easily extractable and stable molecular markers which can be purified rapidly and assessed in bulk by mass spectrometry^7,8^. In previous work, we and others demonstrated the utility of microvesicle proteomics for identifying novel biomarkers for predicting primary graft dysfunction before transplant and for diagnosing cellular and antibody mediated rejection^9–11^. The underlying premise of such an approach is to use a large and relatively understudied biomarker set to improve prediction accuracy and shed insight into the molecular characteristics that accompany either a positive or negative outcome. Analysis of gene sets and pathways that are either enriched or depleted in the clinical setting of interest can lead to non-intuitive and novel findings that underly disease processes^12^. In this study, we focus on the recipient prior to transplant and utilize the microvesicle proteome to identify new biomarkers and the molecular pathways that may predict survival after transplant (Fig. 1).

## Results

### Patient clinical characteristics

The patient cohort in this study was comprised of 88 patients who underwent heart transplantation between 2014 and 2016 at Cedars Sinai Medical Center (n = 43), Pitié Salpêtrière University Hospital (n = 29) and Columbia University Irving Medical Center (n = 16) (Table 1 and Table S1). There were 37 different pre-transplant clinical characteristics across all the patients including survival post-transplant (Table 1). There were 22 deaths (25%), and a maximum follow up of up to ten years (median: 6.5 years) in our cohort (Fig. S1). We found no pre-transplant characteristic significantly associated with patient survival (significance alpha threshold of p-value = 0.05). As a control analysis, we analyzed primary graft dysfunction (PGD) after transplant and found it to significantly associate with patient survival in both univariate and multivariate analyses (logistic regression p-value = 0.019).

**Table 1:** Clinical characteristics.

	Died	Survived	p-value	Multivariate p-value
N	22	66		
**Patient characteristics**				
Age (mean (SD))	57.48 (12.63)	56.28 (11.91)	0.69	0.389
BMI (mean (SD))	26.95 (5.43)	25.36 (4.26)	0.162	0.199
Blood Type (%)			0.054	
A	13 (59.1)	21 (31.8)		0.881
AB	3 (13.6)	5 (7.6)		0.51
B	1 (4.5)	12 (18.2)		0.200
O	5 (22.7)	28 (42.4)		0.062
Donor age (mean (SD))	43.36 (14.74)	39.47 (13.20)	0.248	0.355
Sex = F (%)	10 (45.5)	17 (25.8)	0.142	0.21
History of tobacco use = Y (%)	6 (27.3)	25 (37.9)	0.519	0.885
Diabetes = Y (%)	11 (50.0)	18 (27.3)	0.089	0.383
Cohort (%)			0.115	
Cedar-Sinai	14 (63.6)	29 (43.9)		0.55
Columbia	1 (4.5)	15 (22.7)		0.27
Pitié Salpêtrière	7 (31.8)	22 (33.3)		0.99
**Cardiomyopathy**				
Ischemic = Y (%)	8 (36.4)	24 (36.4)	1	0.268
Non-Ischemic (%)			0.271	
Adriamycin	1 (4.5)	0 (0.0)		1
Amyloid	0 (0.0)	2 (3.0)		1
Chagas	0 (0.0)	1 (1.5)		–
Congenital	1 (4.5)	0 (0.0)		1
Hypertrophic cardiomyopathy	0 (0.0)	1 (1.5)		–
Idiopathic	11 (50.0)	36 (54.5)		–
Myocarditis	0 (0.0)	1 (1.5)		1
Valvular heart disease	1 (4.5)	0 (0.0)		–
Viral	0 (0.0)	1 (1.5)		–
**Transplant factors**				
**PGD = Y (%)**	**20 (90.9)**	**22 (33.3)**	**< 0.001**	**0.019**
Ischemic Time (min (SD))	154.45 (61.18)	165.19 (57.73)	0.459	0.048
Ventricular Assist Device = Y (%)	5 (22.7)	16 (24.2)	1	0.953
**Hemodynamics**				
PA Diastolic (mean (SD)) mmHg	20.05 (8.08)	20.74 (6.98)	0.7	0.092
PA Systolic (mean (SD)) mmHg	45.93 (15.03)	43.49 (13.36)	0.475	0.941
PA Mean (mean (SD)) mmHg	31.78 (8.39)	29.74 (8.77)	0.341	0.687
CVP (mean (SD)) mmHg	10.56 (4.95)	9.44 (5.30)	0.387	0.774
PCWP (mean (SD)) mmHg	21.21 (8.13)	19.52 (8.34)	0.408	0.200
**Lab values**				
Creatinine (mean (SD)) mg/dL	1.32 (0.49)	1.30 (0.98)	0.942	0.160
INR (mean (SD))	1.73 (0.80)	1.50 (0.55)	0.135	0.063
TBili (mean (SD)) mg/dL	0.83 (0.47)	0.87 (0.50)	0.744	0.102
Sodium (mean (SD)) mEq/L	138.16 (4.03)	136.90 (5.06)	0.294	0.489
**Medications**				
Antiarrhythmic Use = Y (%)	15 (68.2)	32 (48.5)	0.175	0.200
Beta Blocker = Y (%)	15 (68.2)	39 (59.1)	0.613	0.143
Inotrope = Y (%)	7 (31.8)	37 (56.1)	0.085	0.176
**Composite scores**				
CVP/PCWP (mean (SD))	0.54 (0.27)	0.51 (0.27)	0.659	0.355
MELD-XI (mean (SD))	7.19 (4.77)	6.89 (4.27)	0.788	0.116

Recipient characteristics at the time of transplant unless otherwise specified. Significance evaluated with a continuity-corrected chi-squared test for categorical characteristics and t-test for continuous characteristics. Variables with a dash contributed to a singular matrix when calculating ordinary least squares multivariable regression and were omitted prior to fitting the model.

*PGD* Primary Graft Dysfunction, *BMI* Body Mass Index, *PA* Pulmonary Artery, *CVP* Central Venous Pressure, *PCWP* Pulmonary Capillary Wedge Pressure, *INR* International Normalized Ratio, *TBili* Total Bilirubin, *MELD-XI* Model for End Stage Liver Disease Score excluding INR.

Significant values are in [bold].

### Microvesicle proteomics

Microvesicles were isolated from pre-transplant serum samples and underwent mass spectrometry analysis in at least triplicate per patient (totaling 322 spectra). Protein expression from each site of collection displayed a non-parametric distribution (Omnibus test of normality p-values ≪ 0.001; Fig. S2). Protein expression was significantly different between each site of collection (Columbia comparison to Cedars, Kolmogorov Smirnov test p-value < 1.19E − 07; Columbia to Paris Kolmogorov Smirnov test p-value = 2.38E − 05; Paris to Cedars, Kolmogorov Smirnov test p-value = 0.008). Of the 681 unique proteins identified, 265 proteins were present in all samples. A final set of 181 proteins was used for the analysis after excluding immunoglobulin proteins and proteins without gene name annotations.

### Prediction of post-transplant survival using pre-transplant clinical and protein markers

**Figure 2:**
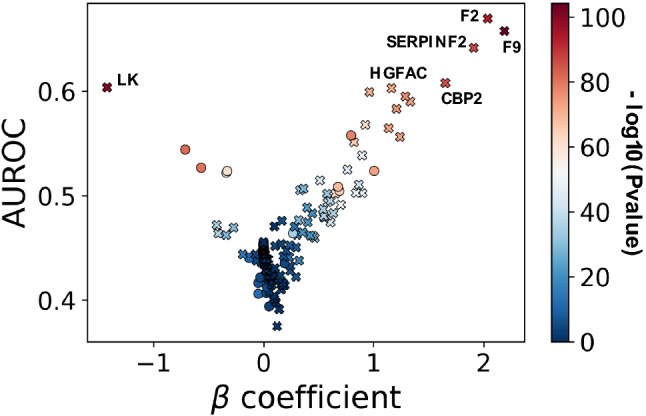
Clinical and protein predictive markers of patient survival after transplant. Post-transplant survival prediction, by Monte Carlo Cross Validation (see “Methods”), of the 181 proteins and 37 binarized clinical characteristics. Protein (crosses) and clinical (circles) marker association (beta coefficient; a measure of influence towards patient survival) versus predictive performance (AUROC). A L1-regularized logistic regression model estimated the association (beta coefficient) of each marker to post-transplant patient survival. The diverging color palette indicates the negative log10 of the feature importance significance or p-value, after Bonferroni correction, from a permutation analysis. The significantly predictive markers with an AUROC > 0.6 are labelled.

**Figure 3:**
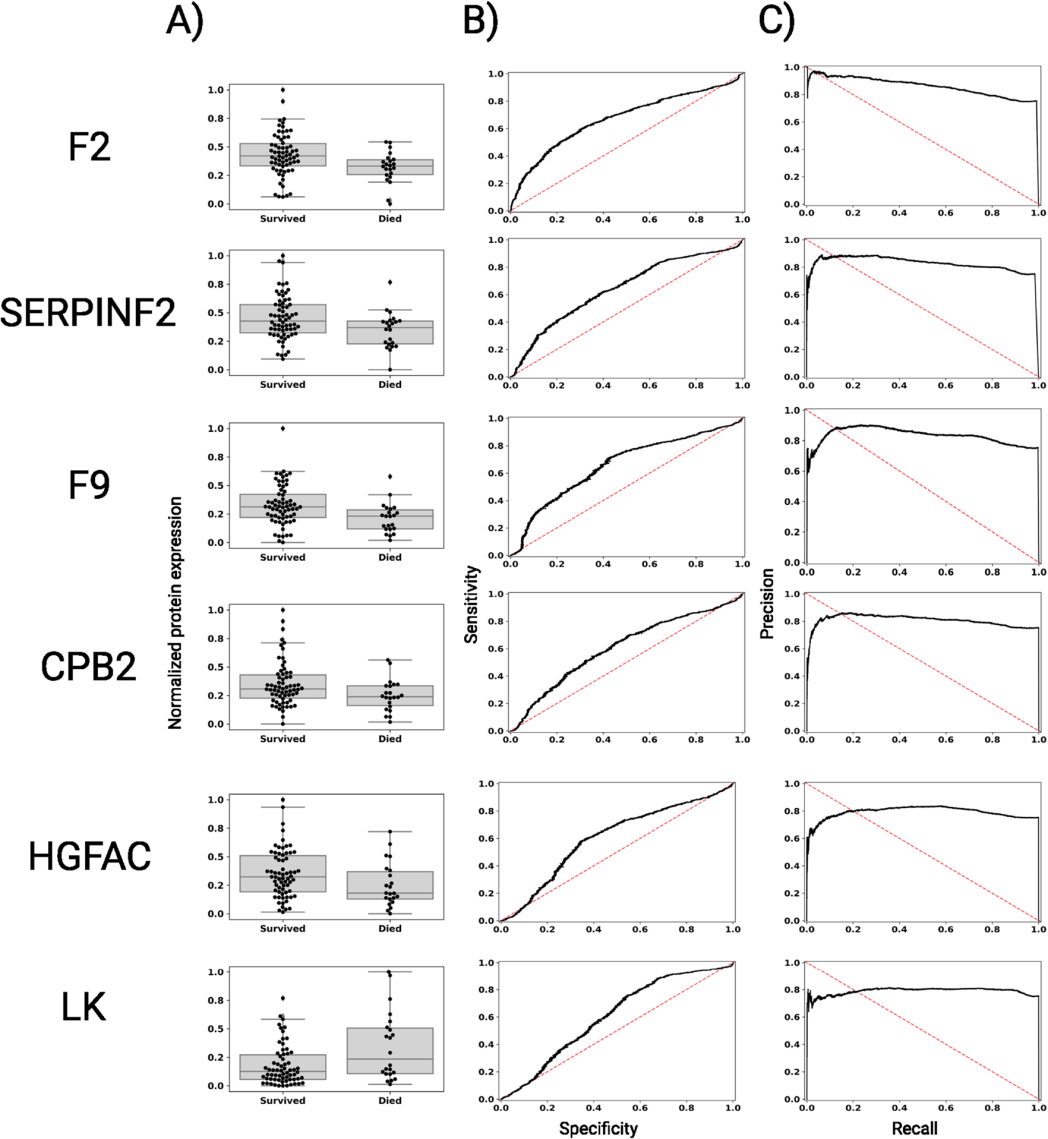
Predictive protein distributions and performance for post-transplant survival. For the highly predictive proteins (AUROC > 0.6), we show (**A**) the maximum-minimum normalized protein distributions for patients (and replicate samples) grouped by patients who survived or died after heart transplant, (**B**) the receiver operating characteristic (ROC) curve between sensitivity and 1-specificity, and (**C**) the precision-recall curve.

We investigated the prediction by pre-transplant protein and clinical markers for post-transplant patient survival. Monte Carlo Cross Validation (MCCV) and permutation analysis was employed to calculate the prediction interval and significance of each clinical and protein marker in predicting patient survival after heart transplant (Fig. 2; See “Methods” for MCCV algorithm). We found that marker prediction varied when accounting for patient site-of-origin (Fig. S3). Eighteen clinical and protein markers were significantly predictive for patient survival after transplant (Table 2; AUROC > 0.5, beta coefficient 95% confidence interval not containing the null association, and permutation beta coefficient interval containing the null association). After adjusting for patient site of origin, 11 clinical and protein markers remained significantly predictive of post-transplant survival (Fig. 2). Increased expression of prothrombin (F2), alpha 2-antiplasmin (SERPINF2), coagulation factor IX (F9), carboxypeptidase 2 (CPB2) and hepatocyte growth factor activator (HGFAC) and decreased expression of low molecular weight kininogen (LK) were most predictive (AUROC > 0.6) of patient survival (Fig. 3).

**Table 2:** Significant markers of post-transplant survival.

	AUROC 2.5%	AUROC	AUROC 97.5%	Beta 2.5%	Beta	Beta 97.5%
**F9**	**0.634**	**0.658**	**0.685**	**1.261**	**2.188**	**3.443**
**F2**	**0.649**	**0.670**	**0.684**	**1.240**	**2.036**	**2.758**
**SERPINF2**	**0.621**	**0.642**	**0.663**	**0.975**	**1.909**	**2.662**
**CPB2**	**0.590**	**0.608**	**0.631**	**0.800**	**1.651**	**2.782**
**ITIH2**	**0.574**	**0.590**	**0.611**	**0.663**	**1.334**	**1.998**
FBLN1	0.569	0.595	0.618	0.443	1.288	2.432
CLEC3B	0.536	0.556	0.579	0.413	1.240	2.057
HPR	0.559	0.583	0.604	0.444	1.207	2.297
**HGFAC**	**0.584**	**0.603**	**0.628**	**0.335**	**1.162**	**2.168**
CD5L	0.543	0.564	0.583	0.265	1.138	2.018
KRT10	0.579	0.599	0.618	0.075	0.963	1.994
FCN2	0.518	0.539	0.563	0.053	0.894	1.753
**Inotrope Therapy**	**0.536**	**0.558**	**0.578**	**0.428**	**0.794**	**1.248**
PF4	0.505	0.525	0.543	0.114	0.762	1.769
**Diabetes**	**0.508**	**0.527**	**0.555**	**− 0.956**	**− 0.566**	**− 0.099**
**Blood type A**	**0.526**	**0.544**	**0.570**	**− 1.016**	**− 0.712**	**− 0.298**
**LK**	**0.585**	**0.604**	**0.628**	**− 2.272**	**− 1.421**	**− 0.445**
*PGD*	*0.706*	*0.723*	*0.744*	− *2.513*	− *2.055*	− *1.726*

Bold values significantly predicted post-transplant survival after adjustment for patient site-of-origin. The positive control, PGD, is highlighted in italics.

*PGD* primary graft dysfunction, *LMW* low molecular weight.

Short and long-term survival after transplant are likely mediated by different underlying mechanisms and it is unknown what distance from a transplant risk can be assessed using pre-transplant proteomics. We performed an analysis to predict 1-year survival post-transplant to compare the predictive profiles between near term (< 1 year) and long term (> 1 year) survival. This did diminish the number of mortality events and thus the power of the analysis as 7 of 22 deaths occurred after one year. SERPINF2, F9, and LK remained significant predictors while F2, CPB2 and HGFAC were no longer predictive (Table 3). This demonstrated that there was some attenuation of prediction performance in several of the proteins when focusing on 1 year survival, though the predictive metrics of those proteins that remained significant were unchanged.

**Table 3:** Comparison of significantly predictive proteins between survival prediction schemes.

	Survival (all-time)	Survival (1-year)	Survival (all-time) with PGD covariate
F2	0.670 [0.649, 0.684]	–	–
SERPINF2	0.642 [0.621, 0.663]	0.651 [0.632, 0.678]	0.826 [0.812, 0.845]
F9	0.658 [0.634, 0.685]	0.675 [0.658, 0.697]	0.842 [0.825, 0.857]
CPB2	0.608 [0.590, 0.631]	–	0.832 [0.818, 0.843]
HGFAC	0.603 [0.584, 0.628]	–	0.793 [0.776, 0.808]
LK	0.604 [0.585, 0.628]	0.678 [0.654, 0.707]	0.804 [0.786, 0.820]

Proteins listed were significantly predictive of all-time survival post-transplant after accounting for site-of-origin. Those proteins that did not meet significance criteria are indicated by ‘–’. Predictive significance criteria were: AUROC > 0.5, beta coefficient 95% confidence interval not containing the null association, and permutation beta coefficient interval containing the null association.

In a secondary control analysis, given its already known association with mortality, we investigated PGD and found it to be a predictive clinical marker (AUROC: 0.723 [0.706, 0.744], Beta coefficient: − 2.06 [− 2.514, − 1.726]) (Table 2, italics). Though this analysis is agnostic to the cause of death, the prevalence of PGD in this cohort does raise the question of whether the predictive performance of the proteins is in some way linked to PGD. Thus, we performed the analysis accounting for PGD status as a covariate. We found all predictive proteins had in fact higher performance (AUROC > 0.71) when accounting for PGD, demonstrating that prediction was not dependent on PGD status (Table 3). We also compared the predictive performance of proteins for survival to PGD and did not find a statistically significant association (Spearman rho coefficient = 0.074, p-value = 0.3, Fig. S4).

### Post-transplant survival differential signature

**Figure 4:**
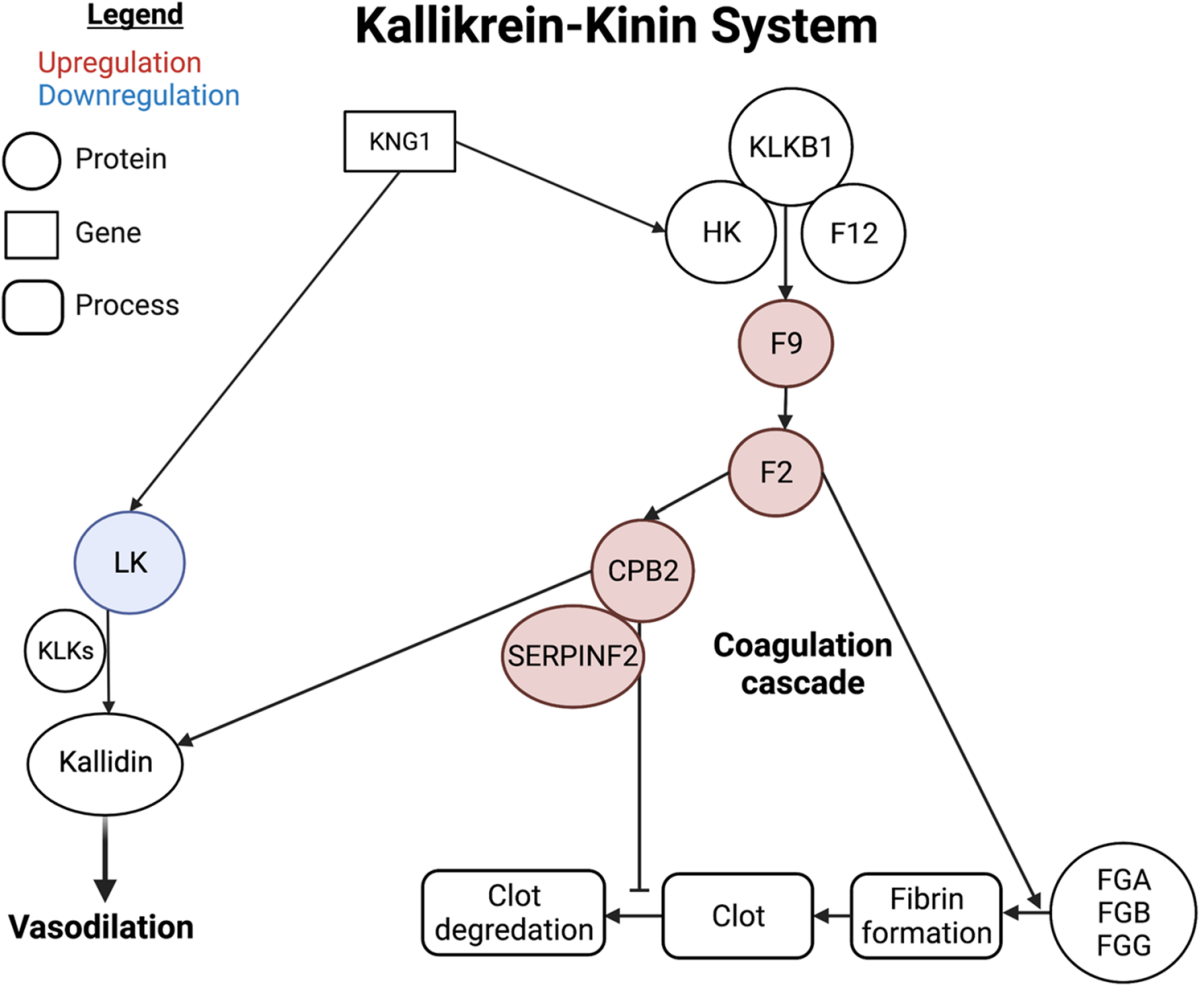
Biological model for post-heart transplant survival regulated by the kallikrein-kinin system (KKS). Our predictive analyses converge on a biological role of the Kallikrein-kinin system (KKS) in patient survival post-transplant. Two pathways of the KKS diverge at kininogen (KNG1) transcription into high molecular weight (HK) or low molecular weight (LK) kininogen. LK is cleaved by tissue kallikreins (KLKs) into kallidin and contributes to vasodilation. HK forms a complex with plasma kallikrein and factor 12 to catalyze coagulation. The waterfall cascade involves factor F9 and factor F2 which, in conjunction with fibrinogens, activate fibrin formation. As clotting is upregulated by the increased expression of predictive proteins, the proteinases CPB2 and SERPINF2 inhibit the degradation of clots. Figure made with Biorender.

We investigated biological pathways associated prior to heart transplant to elucidate putative mechanisms contributing to patient survival. There were 262 proteins expressed in all patients including immunoglobulins to compute a differential protein signature. Immunoglobulins were not significantly different, on average, from non-immunoglobulins across patients (Mann Whitney p-value = 0.264). Gene set enrichment analysis was utilized on differential protein expression and pathways and functions (FDR < 0.2) were found to be enriched for post-transplant survival (Table 4 and Table S2). Enriched pathways associated with survival included platelet activation and the coagulation cascade. Of the predictive proteins with AUROC > 0.6, F2, F9, CPB2, SERPINF2 and LK are all components within the kallikrein-kinin pathway (Fig. 4).

**Table 4:** Significantly enriched pathways for post-transplant patient survival.

	Normalized enrichment score	False discovery rate
Response to elevated platelet cytosolic Ca^2+^_Homo sapiens_R-HSA-76005	4.156	< 0.001
Extracellular matrix organization_Homo sapiens_R-HSA-1474244	4.163	< 0.001
Platelet degranulation_Homo sapiens_R-HSA-114608	4.156	< 0.001
Metabolism of proteins_Homo sapiens_R-HSA-392499	− 3.418	0.116
Platelet activation, signaling and aggregation_Homo sapiens_R-HSA-76002	3.783	0.138
Signal Transduction_Homo sapiens_R-HSA-162582	3.546	0.189

Significance evaluated by phenotype permutation False Discovery Rate < 0.2. Sorted by FDR.

## Materials and methods

### Patient cohorts

The study was designed in accordance with the rules of Good Clinical Practice and with the ethical principles established in the Declaration of Helsinki. The cohort of patients used in this study was previously described^11^, comprising heart transplant patients with and without severe primary graft dysfunction (PGD) using ISHLT criteria^13^ matched by gender and age. Patient serum samples were prospectively recruited at Columbia University Irving Medical Center (Columbia) between 2014 and 2016. Patient serum samples were retrospectively collected from biobanks at Cedars-Sinai hospital (Cedars) and Pitié Salpêtrière University Hospital (Paris). Patients undergoing re-transplant were excluded. For 81 patients, a single serum sample was provided and analyzed. Seven patients from the Paris cohort had two serum samples provided and all expression and prediction analyses averaged the protein quantities of those two samples. Human subjects protocol was approved by the Institutional Review Boards of Columbia University, Cedars Sinai and Pitié Salpêtrière University Hospital and patients provided informed consent. Patient characteristics were collected including demographics, biometrics, labs, medications and hemodynamics. We derived the MELD-XI score for each patient using the formula: 3.78 × ln[serum bilirubin (mg/dL)] + 9.57 × ln[serum creatinine (mg/dL)] + 6.43^13^.

We performed a multivariate logistic regression model to determine significance of each clinical characteristic’s association to patient survival amongst all clinical characteristics. For characteristics missing in less than a third of patients, we imputed the most frequent value or the average value, for binary/categorical and numeric characteristics respectively. The patient cohort table was constructed using custom Python and R scripts using the tableone R package.

### Mass spectrometry analysis

Total microvesicle was isolated from 100 μl of serum using an optimized protocol based on a commercial total microvesicle isolation kit from Life Technologies Inc. (ThermoFisher Total Exosome Isolation from Serum, 4478360), specifically including an incubation step at 4 degrees (Step 3) and a resuspension volume of 25 µl (Step 6). Samples were homogenized using MS-compatible lysis buffer (4 M Urea/50 mM Ammonium bicarbonate/protease inhibitor & phosphatase inhibitor). 20 μg of lysate from each sample was proteolytically cleaved with trypsin and chemically labeled with mass spectrometer detectable quantification reagent, TMT10plex isobaric mass tags separately^14–16^. Sample preparation quality control was performed by TMT labels checking and tryptic digestion efficiency (100 ng of each sample was pooled, desalted, and analyzed by short SPS-MS3 method, and using normalization factor, samples were bulk mixed at 1:1 across all channels). Quality control to check LC–MS performance was performed using Pierce™ HeLa Digest/PRTC Standard (Catalog number: A47997) and Pierce™ TMT11plex Yeast Digest Standard (Catalog number: A40938).

A reference sample was generated by pooling equal amounts of serum microvesicles from each patient to create a common protein library for quantification. Samples were bulk mixed at 1:1 across all channels and bulk mixed samples were fractionated using the Pierce™ High pH Reversed-Phase Peptide Fractionation Kit (Thermo Scientific). Each fraction was dried down in a speed-vac and dissolved in a solution of 2% acetonitrile/2% formic acid. Each fraction was injected in triplicate on Oribitrap Fusion coupled with the UltiMate™ 3000 RSLCnano system (Thermo Scientific). Fractionated peptides were separated from the self-made 25 cm column (Resprosil-C18, 2.4 mm, 25 cm × 75 mm, Dr. Maisch GmbH) at a non-linear flow rate of 300 nl/min using a gradient of 5–30% of buffer B (0.1% (v/v) formic acid, 100% acetonitrile) for 70 min with a temperature of the column maintained at 40 °C during the entire experiment. The full MS spectra were acquired in the Orbitrap Fusion™ Tribrid™ Mass Spectrometer (Thermo Scientific) at a resolution of 120,000. The 10 most intense MS1 ions were selected for MS2 analysis. The isolation width was set at 0.7 Da and isolated precursors were fragmented by CID at a normalized collision energy (NCE) of 35% and analyzed in the ion trap using “turbo” scan speed. Following acquisition of each MS2 spectrum, a synchronous precursor selection (SPS) MS3 scan was collected on the top 10 most intense ions in the MS2 spectrum. SPS-MS3 precursors were fragmented by higher energy collision-induced dissociation (HCD) at an NCE of 60% and analyzed using the Orbitrap. Raw mass spectrometric data were analyzed using Proteome Discoverer 2.2 to perform database search and TMT reporter ions quantification. TMT tags on lysine residues and peptide N termini (+ 229.163 Da) and the carbamidomethylation of cysteine residues (+ 57.021 Da) was set as static modifications, while the oxidation of methionine residues (+ 15.995 Da), deamidation (+ 0.984) on asparagine and glutamine were set as a variable modification. Data were searched against a UniProt human database with peptide-spectrum match (PSMs) and protein-level at 1% FDR. The signal-to-noise (S/N) measurements of each protein were normalized so that the sum of the signal for all proteins in each channel was equivalent to account for equal protein loading. The results obtained from PD2.2 were further analyzed as described below.

### Protein expression analysis

We calculated a differential protein expression signature between survived and expired patient samples, as previously described^11^. The protein association calculated was used as the differential rank statistic for pathway analysis using gene set enrichment analysis (GSEA)^12^.

All the statistical analyses were done in the Python programming language (Python Software Foundation. Python Language Reference, version 3.7. Available at http://www.python.org). The software platform STRING investigated cellular component enrichment of the identified proteins.

The difference in protein expression distributions between the prospective and retrospective cohorts was tested with the Kolmogorov–Smirnov 2-sample test. The protein expression distribution deviation from normality test is from D’Agostino’s and Pearson’s test, where normality of a distribution is rejected at an alpha level p-value of 0.05. Both methods are from the python package *Scipy*. We calculated a differential protein expression signature between survived and expired patient samples. To estimate association of individual protein levels to survival, we calculated L1-regularized logistic regression models for each protein with the sites-of-origin as covariates. We performed 200 bootstraps (samples with replacement) of the models to determine a confidence interval for the protein expression association to survival. The average of the bootstrap distribution for each protein was used as the differential rank statistic.

For 81 patients, a single serum sample was provided and analyzed. Seven patients from the Paris cohort had two serum samples provided, resulting in 95 total samples. We examined whether the additional samples were more correlated in the expression of the 181 proteins. Thus, we computed 95 choose 2 or 4465 pairwise (spearman) correlations across 181 proteins. Only 71 (1.6%) had a spearman correlation over 0.5, where 13 included a technical replicate. The variability in sample expression suggests technical replicates are not likely to inflate protein expression differences for patient survival. For the analysis, we averaged together protein values between the two replicates of the 7 samples resulting in one sample for each of the 88 patients for downstream analysis.

Pathway analysis was conducted using gene set enrichment analysis (GSEA)^12^. The GSEA algorithm employed was from the python package *gseapy* version 0.9.15. The pathway and function gene lists used in our GSEA analysis were ‘GO_Biological_Process_2017b’, 'GO_Molecular_Function_2017b', 'GO_Cellular_Component_2017b', 'Reactome_2016', 'WikiPathways_2019_Human', ‘'KEGG_2019_Human', which are all in the gseapy package hosted on its website (https://amp.pharm.mssm.edu/Enrichr/#stats). The statistics generated by the GSEA algorithm is detailed in the user guide (https://www.gsea-msigdb.org/gsea/doc/GSEAUserGuideFrame.html). Briefly, the Normalized Enrichment Score (NES) provides a gene set enrichment compared to all permutations of the gene set enrichments for the protein expression data. The NES can be interpreted as the gene set enrichment score corrected for the size of the gene set and spurious, un-interesting correlations between the gene sets and the expression dataset. The p-value estimates the probability of seeing an enrichment score as high or higher among the permutation distribution, and the false discovery rate (FDR) estimates the probability that an enrichment score with a given NES is a false positive finding. The leading edge (ledge) genes are the genes from the pathway gene set with the highest impact on the signal generated for the biological process.

### Survival prediction

The prediction scheme, Monte Carlo Cross Validation (MCCV), is comprised of the following steps repeated 200 times^11,17^:

1. Split the data into 85% training and 15% validation sets.
2. Separately normalize, or subtract the sample mean and divide by the sample standard deviation, the training and testing data.
3. Using only the sampled training data, compute tenfold cross validation and choose the top performing model parameters for predicting survival status.
4. Refit the training dataset using the top-prediction model parameters determined in step 3.
5. Predict the survival status of the patients in the yet-to-be-seen validation set using the refit model calculated in step 4.

Specifically, we begin by computing 200 randomized training/validation data splits for the prediction steps outlined above (step 1). We performed normalization (step 2; min–max scaling within the training and validation sets, separately) on the clinical and proteomic data separately for the training and validation data. Within each of the 200 randomized training/validation data splits, we used tenfold cross validation (within the training set only) to optimize model parameters and perform feature selection (step 3). Using the chosen parameters and features, we then train on the entire training set (step 4) and use this model to predict survival status on the validation set (step 5). The survival prediction probabilities were compared to the true survival status to compute the area under the receiver operating characteristic curve (AUROC), and other metrics. The AUROC values reported in this paper are calculated using the validation set patient probabilities. Bootstrapping analysis on the validation patient probabilities (N = 50 samples with replacement) resulted in a population distribution for prediction performances, and feature importance (beta coefficient) was extracted within each bootstrap before prediction on the validation set. A permutation analysis was similarly performed, with random labeling of survival status in patients, to generate and test from a distribution of prediction metrics from random survival assignment. Comparison of the bootstrap and permutation prediction distributions allows for prediction and feature importance comparisons between real and randomly distributed data while accounting for over-fitting during these prediction tasks. We evaluated the significance of each marker to predict patient survival by comparing the 200 feature importance values from the bootstrap and the permutation prediction distributions. The p-values generated in this comparison represent protein marker prediction in our cohort compared with random patient survival. We tested differences in the bootstrap and permutation distributions with the 2-sample Kolmogorov–Smirnov test.

We note that our methodology permits prediction of death as well as survival. In this case, the machine learning models produce higher probabilities for expired patients which result in AUROC values less than 0.5, which is often regarded as a random prediction. Our MCCV methodology samples these patient probabilities to derive an AUROC performance metric and confidence interval. The calculated marker performances are representative of the model’s confidence in predicting patient survival.

We performed several binary schemes to evaluate our predictive results. The main analysis included the binary prediction of post-transplant survival where the patient did not die after transplantation (all-time survival). We also included covariates in the logistic regression model, such as site-of-origin and post-transplant PGD indicators. Finally, we predicted post-transplant survival within 1-year where patients were labelled as survived as long as they did not die within 1 year of heart transplantation.

## Discussion

In an imbalanced system of limited organ availability and increasing need, risk prediction has the potential to improve organ utilization. Assessment of recipient candidacy is a critical part of all transplant evaluations and involves a thorough analysis of both the recipient and the donor. Inherent in this process is an understanding of what generates that risk and importantly, whether that risk can be modified. The premise of this study is to focus on the pre-transplant recipient and explore the circulating molecular markers using exosome proteomics with the goal of discovering new post-transplant risk factors. Underlying this is the hypothesis that some amount of post-transplant risk is predicated on molecular factors within the recipient that are present at the time of transplant and can be estimated reliably prior to transplant.

We used our Monte-Carlo Cross Validation technique to estimate the variance in post-transplant survival amongst a modest cohort of patients with varying clinical characteristics. In our survival analysis, several proteins were identified as predictive of post-transplant mortality and nearly all were involved in the KKS. The KKS is an enzymatic cascade that produces a number of different bioreactive small molecules involved in inflammation, vasodilation and coagulation and is antagonized by the renin-angiotensin system^18^. At the initiation of the cascade, kininogen is converted into its active kinins including vasoreactive small molecules bradykinin (from high molecular weight kininogen) and kallidin (from low molecular weight kininogen)^19^. Both bradykinin and kallidin signal through the bradykinin receptor and cause vasodilation and the release of nitric oxide and prostacyclins^20^. Kallikrein also activates the intrinsic cascade by proteolytic activation of coagulation factor XII^21^. Peptidases such as angiotensin converting enzymes (ACE) downregulate this pathway by proteolytic degradation of bradykinin and kallidin^22,23^.

Pathway analysis from differential protein expression associated with survival was shifted towards higher levels of components in the coagulation cascade and away from vasoreactive signaling with low molecular weight kininogen. Multiple components of the coagulation cascade were found to be upregulated. Factor IX and Factor II (thrombin) are key enzymes of the intrinsic and common pathways respectively. SERPINF2 or alpha-2-antiplasmin is a serine protease inhibitor that cleaves and inactivates plasmin, an enzyme responsible for the dissolution of fibrin-based clotting^24^. Carboxypeptidases such as CPB2 are activated by thrombin and both inactivate plasmin stabilizing fibrin clots and inhibit inflammations likely through metabolization of complement and pro-inflammatory cytokines^25–28^. In contrast to the elevated levels of these pro-coagulation components, diminished levels of low molecular weight kininogen predicted survival. These results raise the possibility of a pro-inflammatory and vasodilatory state prior to transplant among patients who go on to have a poor outcome.

The transplant patients in this study were comprised for a cohort initially matched for primary graft dysfunction as previously reported^11^. This enriched the mortality of the cohort but at the same time potentially introduced PGD as a confounder given PGD’s association with mortality. When we use PGD as a covariate in our analyses, prediction is improved for most protein markers and overall, there is no significant correlation between prediction metrics for PGD and survival in our analyses. The underlying cause of PGD is not known and its connection to mortality is not mechanistically understood. Thus, the molecular processes lead to PGD may be shared with overall mortality and in our previous analysis of PGD prediction, we identified plasma kallikrein, a component of the KKS, as predictive of PGD. Plasma kallikrein however was not predictive of survival in this analysis. Furthermore, out of the 16 proteins predictive of PGD and the 7 proteins predictive of survival after transplant, only F2 was significant in both analyses. Still, there remains the possibility that dysregulation of the KKS prior to transplant is a unified predictor for poor post-transplant outcomes and how PGD and survival are linked molecularly remains to be delineated.

There are several limitations to the study. Foremost is the small sample size which reduces our power of detecting significant associations and predictions. Through proteomics we are able to construct a high dimensional data set with only a modest number of patients however it remains to be seen whether these predictive proteins are applicable to the larger transplant population. Though the training and testing set were derived from three different institutions, further external validation and prospective analysis of the KKS and individual component biomarkers prior to transplant is required. We also do not take into account donor or organ characteristics that may influence risk. Pre-transplant risk stratification is often done well in advance of a transplant. However, the ability to risk stratify with novel protein biomarkers raises the possibility of generating usable donor-recipient risk pairing in which matching an organ takes into account molecular compatibility anchored in both mechanistic and empiric data. This will require careful prospective and broad biomarker characterization of donors and recipient pairs along with longitudinal outcomes. Lastly, the mass spectrometry results are semi quantitative and thus are not able to definitively provide absolute concentrations of these biomarkers per sample. Utilization of these results will require further characterization and validation of these biomarkers using facile quantitative measurement such as through ELISA that can be more readily deployed in the clinical setting.

In summary, this study expands the pool of potential transplant biomarkers associated with transplant survival using microvesicle proteomics. We found SERPINF2, F9, and LK were able to predict survival independent of PGD status with AUROC > 0.6. Pathway analysis revealed that survival was associated with platelet activation and shift in the kallikrein kinin system away from vasodilation and towards coagulation. Prospective studies investigating the KKS prior to transplant are needed to validate these findings with the goal improving transplant outcomes.

## Supplementary Information

Supplementary Information.

## Data Availability

All code for the analyses can be found on the following website: https://github.com/ngiangre/kng1_analysis. We included a versioned release of our code at https://doi.org/10.5281/zenodo.6969703 and at the https://github.com/ngiangre/kng1_analysis/tree/2.0.0. The mass spectrometry proteomics data have been deposited to the ProteomeXchange Consortium via the PRIDE partner repository with the dataset identifier PXD031426.
