# Supplementary material for: Alterations in the kallikrein-kinin system predict death after heart transplant
Source: Sci Rep. 2022 Aug 19;12:14167. doi: 10.1038/s41598-022-18573-2 (PMC9391369; doi:10.1038/s41598-022-18573-2)
Supplement: Supplementary file 1 — Supplementary Information. [file 41598_2022_18573_MOESM1_ESM.docx]

**Supplementary Material**

**Title: Alterations in the kallikrein-kinin system predicts death after heart transplant**

**Authors:** Nicholas P. Giangreco M.Phil.^1^, Guillaume Lebreton M.D.^2^, Susan Restaino M.D.^3^, Maryjane Farr M.D.^4^, Emmanuel Zorn Ph.D.^5^, Paolo C. Colombo M.D.^3^, Jignesh Patel M.D. Ph.D.^6^, Rajesh Kumar Soni Ph.D.^7^, Pascal Leprince M.D.^2^, Jon Kobashigawa M.D.^6^, Nicholas P. Tatonetti Ph.D.^1,8^, Barry M. Fine M.D. Ph.D.^3†*^

**Figure and Table Legends**

Figure S1. Time to patient death post-heart transplant.

Figure S2. Exosome protein expression distributions for the patient cohorts.

Figure S3. Comparison of marker prediction for post-transplant survival when accounting for patient site-of-origin.

Figure S4: Correlation between prediction of PGD and survival for protein markers

Table S1. Baseline clinical characteristics.

Table S2. Significantly enriched pathways for post-transplant patient death.

| **Table S1: Baseline clinical characteristics**  Recipient characteristics at the time of transplant unless otherwise specified. Significance evaluated with a continuity-corrected chi-squared test for categorical characteristics and t-test for continuous characteristics. | | | | | |
| --- | --- | --- | --- | --- | --- |
|  |  | **Cedar-Sinai** | **Columbia** | **Pitié Salpêtrière** | **P-value** |
|  | **N** | **43** | **16** | **29** |  |
| **Patient characteristics** | **Age (mean (SD))** | **57.95 (12.76)** | **56.50 (10.28)** | **54.60 (11.91)** | **0.517** |
|  | **BMI (mean (SD))** | **25.49 (4.96)** | **28.10 (3.58)** | **24.86 (4.22)** | **0.065** |
|  | **Blood Type (%)** |  |  |  | **0.687** |
|  | **A** | **17 (39.5)** | **6 (37.5)** | **11 (37.9)** |  |
|  | **AB** | **4 (9.3)** | **3 (18.8)** | **1 (3.4)** |  |
|  | **B** | **5 (11.6)** | **2 (12.5)** | **6 (20.7)** |  |
|  | **O** | **17 (39.5)** | **5 (31.2)** | **11 (37.9)** |  |
|  | **Donor Age (mean (SD))** | **36.49 (12.21)** | **38.50 (12.18)** | **47.38 (14.05)** | **0.002** |
|  | **Sex = F (%)** | **15 (34.9)** | **2 (12.5)** | **10 (34.5)** | **0.219** |
|  | **History of Tobacco Use = Y (%)** | **2 (4.7)** | **11 (68.8)** | **18 (62.1)** | **<0.001** |
|  | **Diabetes = Y (%)** | **12 (27.9)** | **7 (43.8)** | **10 (34.5)** | **0.504** |
|  | **Survived = Y (%)** | **29 (67.4)** | **15 (93.8)** | **22 (75.9)** | **0.115** |
| **Cardiomyopathy** | **Ischemic = Y (%)** | **12 (27.9)** | **8 (50.0)** | **12 (41.4)** | **0.231** |
|  | **Non-Ischemic (%)** |  |  |  | **0.651** |
|  | **Adriamycin** | **12 (27.9)** | **8 (50.0)** | **12 (41.4)** |  |
|  | **Amyloid** | **1 (2.3)** | **0 (0.0)** | **0 (0.0)** |  |
|  | **Chagas** | **2 (4.7)** | **0 (0.0)** | **0 (0.0)** |  |
|  | **Congenital** | **0 (0.0)** | **1 (6.2)** | **0 (0.0)** |  |
|  | **Hypertrophic cardiomyopathy** | **1 (2.3)** | **0 (0.0)** | **0 (0.0)** |  |
|  | **Idiopathic** | **1 (2.3)** | **0 (0.0)** | **0 (0.0)** |  |
|  | **Myocarditis** | **23 (53.5)** | **7 (43.8)** | **17 (58.6)** |  |
|  | **Valvular Heart Disease** | **1 (2.3)** | **0 (0.0)** | **0 (0.0)** |  |
|  | **Viral** | **1 (2.3)** | **0 (0.0)** | **0 (0.0)** |  |
| **Transplant factors** | **PGD = Y (%)** | **21 (48.8)** | **8 (50.0)** | **13 (44.8)** | **0.927** |
|  | **Ischemic Time (minutes (SD))** | **148.33 (65.49)** | **178.36 (44.66)** | **174.79 (50.02)** | **0.081** |
|  | **Ventricular Assist Device = Y (%)** | **8 (18.6)** | **11 (68.8)** | **2 (6.9)** | **<0.001** |
| **Hemodynamics** | **PA Diastolic (mean (SD)) mmHg** | **19.85 (6.35)** | **15.62 (7.72)** | **24.35 (6.37)** | **<0.001** |
|  | **PA Systolic (mean (SD)) mmHg** | **41.37 (12.08)** | **36.81 (12.59)** | **52.17 (13.21)** | **<0.001** |
|  | **PA Mean (mean (SD)) mmHg** | **29.32 (6.93)** | **24.00 (9.14)** | **35.07 (8.31)** | **<0.001** |
|  | **CVP (mean (SD)) mmHg** | **10.54 (5.27)** | **7.00 (5.79)** | **10.00 (4.39)** | **0.062** |
|  | **PCWP (mean (SD)) mmHg** | **18.99 (7.16)** | **15.38 (10.29)** | **23.87 (7.06)** | **0.002** |
| **Lab values** | **Creatinine (mean (SD)) mg/dL** | **1.41 (1.21)** | **1.28 (0.32)** | **1.18 (0.32)** | **0.54** |
|  | **INR (mean (SD))** | **1.47 (0.56)** | **1.71 (0.73)** | **1.60 (0.66)** | **0.387** |
|  | **TBili (mean (SD)) mg/dL** | **0.75 (0.29)** | **0.53 (0.31)** | **1.21 (0.60)** | **<0.001** |
|  | **Sodium (mean (SD)) mEq/L** | **136.46 (4.16)** | **140.31 (5.16)** | **136.62 (5.07)** | **0.016** |
| **Medications** | **Antiarrhythmic Use = Y (%)** | **27 (62.8)** | **7 (43.8)** | **13 (44.8)** | **0.225** |
|  | **Beta Blocker = Y (%)** | **25 (58.1)** | **14 (87.5)** | **15 (51.7)** | **0.051** |
|  | **Inotrope = Y (%)** | **23 (53.5)** | **5 (31.2)** | **16 (55.2)** | **0.25** |
| **Composite Scores** | **CVP/PCWP (mean (SD))** | **0.57 (0.29)** | **0.52 (0.29)** | **0.44 (0.21)** | **0.107** |
|  | **MELD (mean (SD))** | **13.47 (5.18)** | **14.44 (5.23)** | **14.31 (4.50)** | **0.702** |
|  | **Radial Score (mean (SD))** | **2.51 (1.03)** | **2.19 (1.28)** | **2.38 (1.27)** | **0.626** |
| Abbreviations: Primary Graft Dysfunction, PGD; Body Mass Index, BMI; Pulmonary Artery, PA; Central venous pressure, CVP; Pulmonary capillary wedge pressure, PCWP; International Normalized Ratio, INR; Total bilirubin, TBili; Model for End Stage Liver Disease Score, MELD | | | | | |

**Figure S1: Time to patient mortality post-heart transplant.**


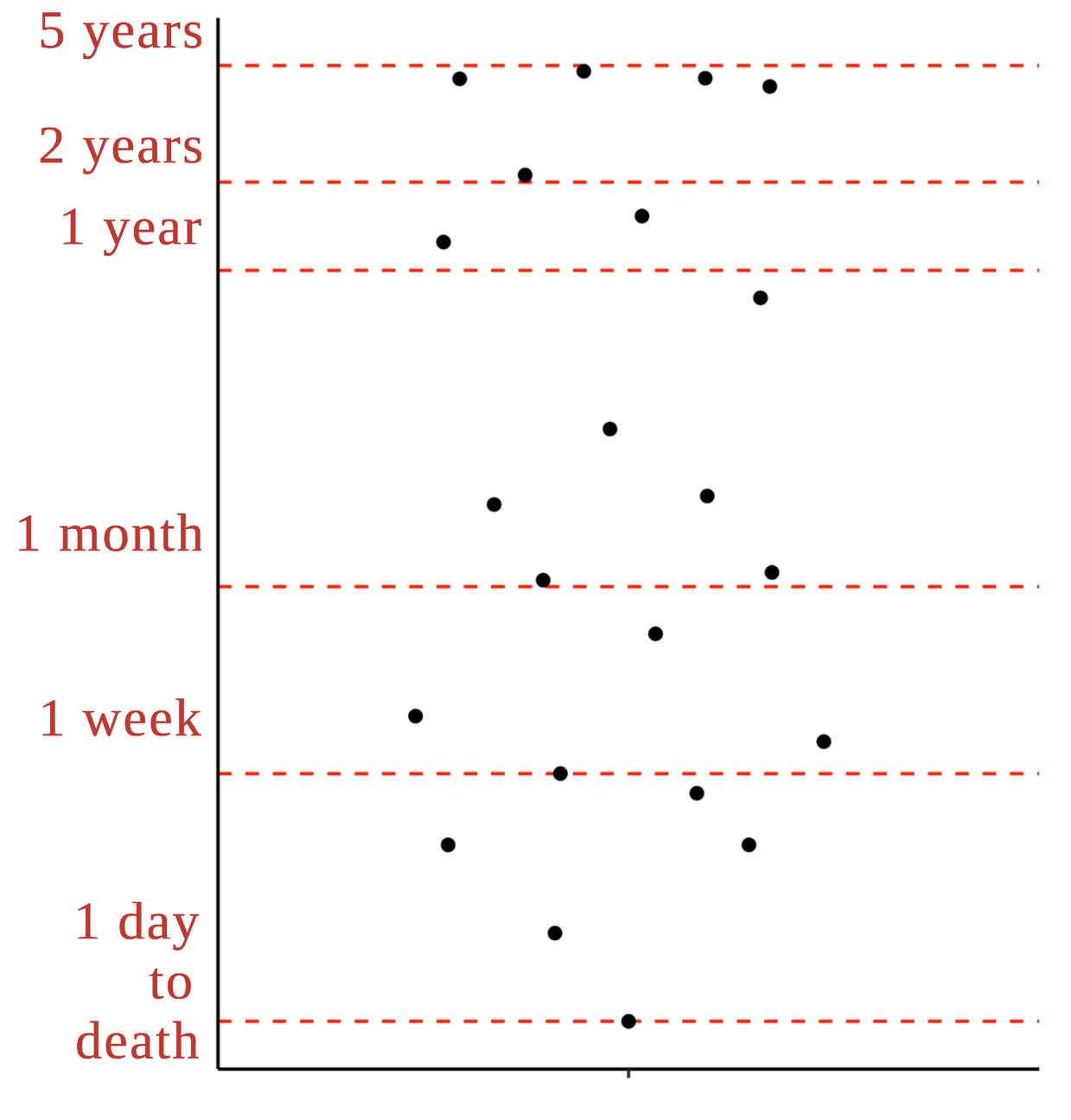


**Figure S2. Exosome protein expression distributions for the patient cohorts.** Protein expression distribution of each patient cohort. The individual patient expression distributions in each cohort are superimposed to represent each patient’s individual contribution to the whole cohort protein expression distribution.

**Figure S3. Comparison of marker prediction for post-transplant survival when accounting for patient site-of-origin.** Comparison between survival prediction A) performance (AUROC) and B) association (beta coefficient) of clinical and protein markers when including (x-axis) and not including (y-axis) covariate adjustment (i.e. site-of-origin).

**Figure S4: Correlation between prediction of PGD and survival for protein markers**

Comparison between survival prediction performance (AUROC) on the X axis PGD prediction performance (AUROC) on the Y axis. Spearman rho coefficient=0.074, p-value=0.3


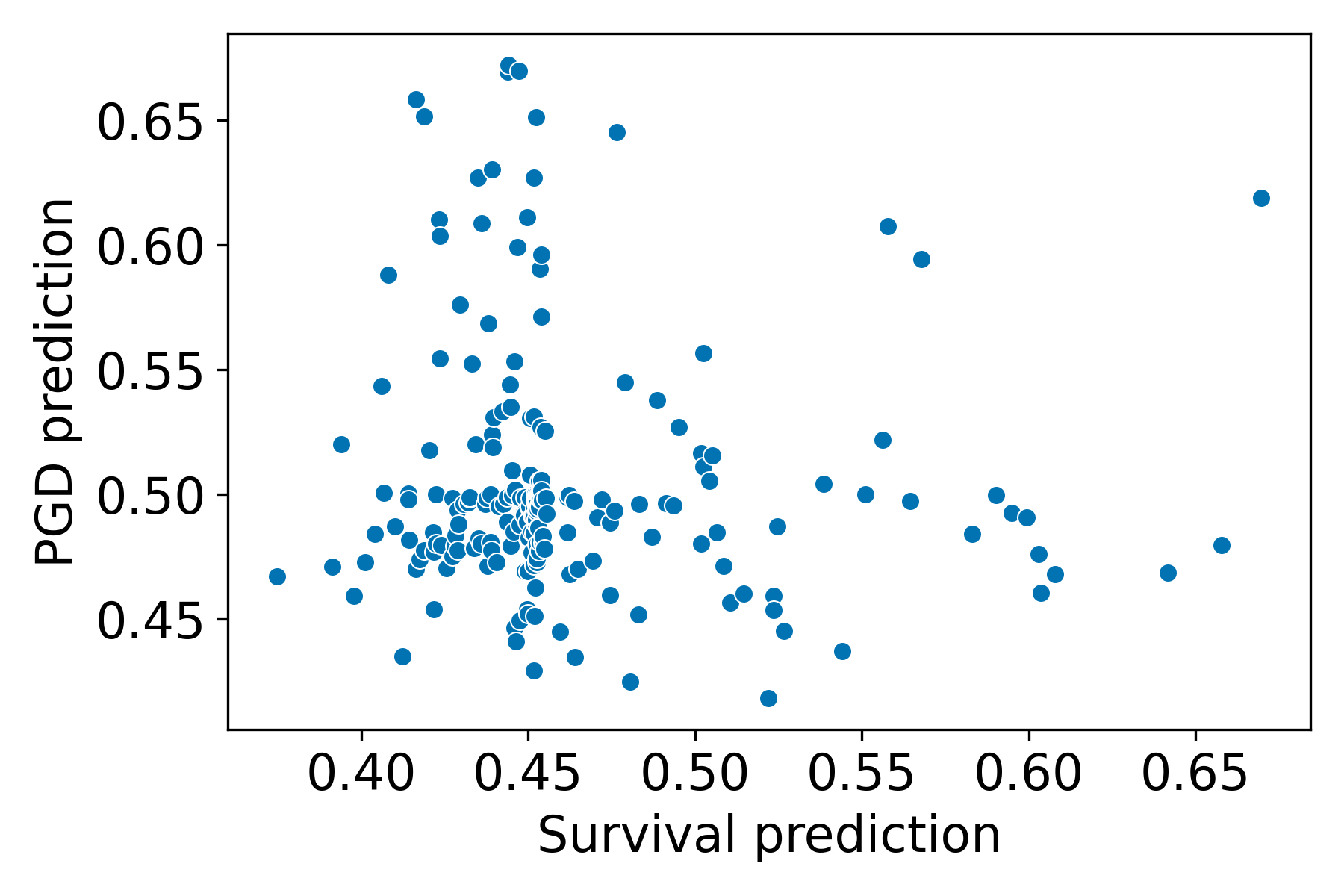


| **Table S2: `Significantly enriched pathways for post-transplant patient survival.**  Significantly enriched pathways by gene set permutation. Sorted by FDR. | | |
| --- | --- | --- |
|  | **Normalized Enrichment Score** | **False Discovery Rate** |
| Complement and Coagulation Cascades WP558 | 1.575 | 0.065 |
| Metabolism of proteins_Homo sapiens_R-HSA-392499 | 1.684 | 0.108 |
| calcium ion binding involved in regulation of cytosolic calcium ion concentration (GO:0099510) | 1.511 | 0.122 |
| Formation of Fibrin Clot (Clotting Cascade)_Homo sapiens_R-HSA-140877 | 1.731 | 0.140 |
| calcium ion sensor activity (GO:0061891) | 1.521 | 0.153 |
| sarcoplasmic reticulum lumen (GO:0033018) | 1.419 | 0.155 |
| cortical endoplasmic reticulum lumen (GO:0099021) | 1.44 | 0.161 |
| serine-type endopeptidase activity (GO:0004252) | 1.55 | 0.184 |
